# Preventing OsteoPorosis in Spinal Cord Injury (POPSCI) Study—Early Zoledronic Acid Infusion in Patients with Acute Spinal Cord Injury

**DOI:** 10.1007/s00223-024-01292-3

**Published:** 2024-09-25

**Authors:** Shejil Kumar, Jean Doyle, Cameron Wood, Roxana Heriseanu, Gerard Weber, Lianne Nier, James W. Middleton, Lyn March, Roderick J. Clifton-Bligh, Christian M. Girgis

**Affiliations:** 1 Endocrinology Department, Royal North Shore Hospital, Sydney, Australia; 2 Faculty of Medicine & Health, University of Sydney, Sydney, Australia; 3 Endocrinology Department, Westmead Hospital, Sydney, Australia; 4 Chemical Pathology Department, Royal North Shore Hospital, Sydney, Australia; 5 Royal Rehab Group, Sydney, Australia; 6 Spinal Cord Injuries Unit, Royal North Shore Hospital, Sydney, Australia; 7 John Walsh Centre for Rehabilitation Research, Northern Sydney Local Health District, St Leonards, Sydney, Australia; 8 Rheumatology Department, Royal North Shore Hospital, Sydney, Australia; 9 Institute of Bone and Joint Research, Kolling Institute of Medical Research, Sydney, Australia; 10 Cancer Genetics Laboratory, Kolling Institute of Medical Research, Sydney, Australia

**Keywords:** Osteoporosis, Spinal cord injury, Zoledronic acid, Bone mineral density, Acute phase reaction

## Abstract

Accelerated sub-lesional bone loss is common in the first 2–3 years after traumatic spinal cord injury (TSCI), particularly in the distal femur and proximal tibia. Few studies have explored efficacy of antiresorptives for acute bone loss prevention post-TSCI, with limited data for knee bone mineral density (BMD) or beyond two years follow-up. An open-label non-randomized study was performed at Royal North Shore Hospital and Royal Rehab Centre, Sydney between 2018 and 2023. An ‘acute interventional cohort’ (*n* = 11) with TSCI (duration ≤ 12-weeks) received a single infusion of 4 mg zoledronic acid (ZOL) at baseline. A ‘chronic non-interventional cohort’ (*n* = 9) with TSCI (duration 1–5-years) did not receive ZOL. All participants underwent baseline and 6-monthly blood tests (including CTx and P1NP) and 12-monthly DXA BMD scans (including distal femur and proximal tibia). Participants were predominantly Caucasian and male (mean age 38.4 years). At baseline, the ‘acute’ cohort had higher serum CTx, P1NP and sclerostin concentrations, while the ‘chronic’ cohort had lower left hip and knee BMD. Majority with acute TSCI experienced an acute phase reaction after ZOL (9/11; 82%). In the acute cohort, left hip BMD fell by mean ~ 15% by 48 months. Left distal femoral and proximal tibial BMD declined by mean ~ 6–13% at 12 months and ~ 20–23% at 48 months, with a tendency towards greater BMD loss in motor-complete TSCI. A single early ZOL infusion in acute TSCI could not attenuate rapidly declining hip and knee BMD. Prospective controlled studies are required to establish the optimal strategy for preventing early bone loss after acute TSCI.

**Supplementary Information:**

The online version contains supplementary material available at 10.1007/s00223-024-01292-3.

## Introduction

Traumatic spinal cord injury (TSCI) is a devastating event. In the past this predominantly affected young men most commonly due to motor vehicle accidents, however, more recently there has been a demographic shift towards a bimodal age distribution related to older people sustaining falls [1]. TSCI is associated with numerous long-term complications, including bone mineral density (BMD) loss and subsequent increased fracture risk [2, 3]. After TSCI, patients typically experience a *biphasic* pattern of BMD loss, with early rapid losses in the first 2–3 years followed by a slower rate of ongoing decline [4, 5]. Bone loss following TSCI predominantly affects *sub-lesional* areas, including the total hip and femoral neck although most profound loss is seen in regions surrounding the knee, including the distal femur and proximal tibia (up to 50% lost in first 3 years), while lumbar spine BMD is preserved [6, 7]. Marked early disturbances in skeletal homeostasis after TSCI predispose to a lifetime increased risk for fracture, with long-term fracture prevalence up to 50% [8, 9]. The distal femur and proximal tibia account for over two-thirds of fragility fractures during chronic SCI, while hip fractures are uncommon and vertebral fractures exceedingly rare [6, 10, 11]. Lower limb fractures are associated with significant morbidity, including prolonged hospitalisation, pressure injuries, fracture non-union and are independently associated with increased mortality risk amongst veteran cohorts [12, 13].

Risk factors for BMD loss and increased fracture risk following TSCI include extent of injury (i.e., complete or incomplete motor loss below lesion) and duration since injury, with an *inflection point* 3–5 years after TSCI beyond when fracture rates begin to increase [3, 4, 9, 10]. The underlying mechanisms driving rapid early bone losses after TSCI are not fully elucidated [2, 5, 14]. Early studies suggested an exaggerated state of bone resorption in the acute phase, and more recent evidence indicates acute immobilisation may trigger dysregulation of osseous Wnt/beta-catenin and RANKL signalling pathways, which drive bone formation and bone resorption, respectively [2, 5].

Given the distinct skeletal phenotype of TSCI-related osteoporosis, studies are needed to specifically assess efficacy and durability of osteoporosis pharmacotherapy in this context, and particularly whether early treatment initiation prevents rapid profound bone loss early after TSCI. Few small, controlled studies have assessed antiresorptive efficacy in preventing bone loss in acute TSCI, most commonly using a single infusion of zoledronic acid (ZOL). Results have been disappointing with modest prevention of BMD loss at the hip and conflicting results at the knee [15, 16]. However, only a small proportion of studies have investigated longitudinal knee BMD response to ZOL, despite being the most clinically relevant site in TSCI-related osteoporosis. Studies have mostly been limited to 1 year follow-up with none assessing durability of response beyond 2 years.

Hence, the POPSCI (Preventing OsteoPorosis in Spinal Cord Injury) study aimed to perform early assessment of musculoskeletal parameters in patients with acute TSCI and investigate longitudinal changes over 4 years in BMD (including proximal tibia and distal femur) and serum bone turnover markers to assess extent and durability of response to a single infusion of ZOL.

## Aims

We hypothesised that patients with acute TSCI would have lower BMD values, and more elevated markers of bone turnover at baseline compared with chronic TSCI patients, and that a single infusion of ZOL in acute TSCI patients would be unable to fully prevent BMD loss at the hip and particularly at the knee over a 4-year follow-up period. The POPSCI study included four aims: (1) to assess longitudinal changes over 4-years in BMD (including regions surrounding the knee) and serum bone turnover markers in (a) patients with acute TSCI in response to a single infusion of ZOL, and (b) in patients with chronic TSCI who did not receive ZOL, (2) to compare baseline bone health parameters including serum and densitometry measurements between acute and chronic TSCI cohorts, and (3) to establish an institutional protocol for measuring knee BMD and assess its precision in patients with TSCI.

## Methods

### Study Design and Participants

We conducted a prospective open-label non-randomised study investigating early administration of intravenous ZOL after acute TSCI and longitudinal effects on BMD and bone turnover outcomes. The study included two separate cohorts. The ‘***acute interventional cohort’*** included patients aged ≥ 18 years who sustained acute TSCI ≤ 12 weeks prior to recruitment and were admitted to Royal North Shore Hospital or Royal Rehab, Sydney (tertiary SCI referral centres) between November 2018 and June 2022. The ‘***chronic non-interventional cohort’*** included patients sustaining TSCI within the last 1–5 years and being followed up by Spinal Rehabilitation Physicians at either site. Participants were excluded if they had non-traumatic SCI, previous antiresorptive use, prior hypersensitivity to ZOL or vitamin D formulations, uncorrected vitamin D deficiency (< 50 nmol/L), impaired renal function (eGFR < 30 mL/min/1.73m^2^), malignancy in the past 5-years other than non-melanoma skin cancer or cervical/breast ductal carcinoma in-situ, or pregnant or lactating women.

### Study Assessments and Follow-up

**Fig. 1:**
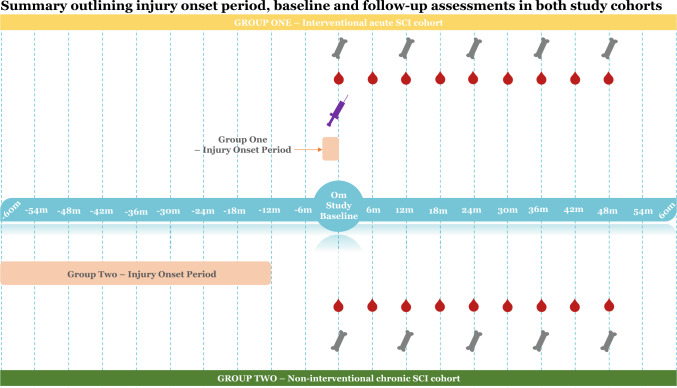
This figure summarises the study procedures and follow-up duration for the two cohorts. The interventional acute cohort included patients who sustained a traumatic SCI within 12-weeks prior to recruitment and underwent an infusion of zoledronic acid at baseline (purple syringe). The non-interventional chronic cohort included patients who suffered traumatic SCI within 1–5 years prior to recruitment and did not receive zoledronic acid. All patients had baseline and 6-monthly serum collected and baseline and 12-monthly DXA scans during 4-year follow-up

Written informed consent was obtained from all participants prior to any study procedures. At baseline study visit, demographic, patient- and injury-related data were obtained via electronic medical record review and patient assessment including age, sex, ethnicity, weight (kg), height (cm) determined by supine length, body mass index (kg/m^2^), current alcohol intake (yes/no) and cigarette smoking (yes/no), time since injury (days), level of injury (cervical vs thoracic vs lumbar) and extent of injury (according to American Spinal Injury Association Impairment Scale (AIS) grades, whereby AIS grades A–B represent complete motor impairment below the lesion and AIS grades C–D incomplete motor impairment). All participants underwent fasting serum collection and dual-energy X-ray absorptiometry (DXA) BMD and whole-body composition scan at baseline study visit (prior to ZOL in the acute cohort). Study visits were 6-monthly, with blood tests repeated 6-monthly and DXA scans annually during 4-year follow-up (Fig. 1). Ambulatory status was assessed by interview and physical examination every 6-months.

### Interventions

After baseline studies were completed, participants in the ‘acute interventional cohort’ received a single dose of 4 mg intravenous ZOL on the same day in the outpatient infusion unit over 15–30 min according to standard manufacturer procedures, while participants in the ‘chronic non-interventional cohort’ did not receive ZOL. Participants receiving ZOL were counselled regarding risk of acute phase reactions (APRs) and advised to administer oral paracetamol 1000 mg up to four times daily commencing the day of infusion and for up to 3-days thereafter. Patients in the acute cohort were reviewed 1–2 days after receiving ZOL to identify symptoms and signs of APR defined as the presence of any of the following within the first 3 days post-infusion: fever (≥ 38.0 °C) or rigors, musculoskeletal symptoms (pain, discomfort), gastrointestinal disturbance, eye inflammation or general symptoms (fatigue, headache) [17]. Presence, severity (grade I–V defined according to CTCAE v5.0), duration (days) and treatment of APRs were recorded prospectively.

### Laboratory Procedures

Fasting serum blood samples for musculoskeletal and hormonal assessment included: full blood count (FBC), liver enzymes including total alkaline phosphatase (ALP), albumin-corrected calcium, magnesium and phosphate, renal function (creatinine, eGFR), intact parathyroid hormone (iPTH), 25-hydroxyvitamin D_3_ (25OHD_3_), luteinising hormone (LH), testosterone in men and oestradiol in women, the bone resorption marker C-terminal telopeptide of type 1 collagen (CTx), the bone formation marker procollagen type 1 N-propeptide (P1NP), sclerostin and myostatin concentrations. Serum β-HCG was also performed for women of childbearing age prior to administration of ZOL and before any DXA assessments. Blood was centrifuged for 15 min at 3500 g with serum then aliquoted and stored at – 80 °C until assays performed in batches. All biochemical analyses (unless otherwise specified) were conducted using the Abbott Architect C16000/I2000sr, in the Endocrinology Research Laboratory, Royal North Shore Hospital, Sydney by senior medical laboratory scientists. Serum sclerostin concentrations were measured using the DiaSorin LIAISON® XL chemiluminescent immunoassay (CLIA) [18]. CTx and P1NP were performed using the Roche Cobas Elecsys E601 automated immunoassay analyser. Myostatin concentrations were measured using the sandwich Quantikine ELISA GDF-8/Myostatin Immunoassay (R&D Systems Inc., Minneapolis, MN, United States) according to manufacturer instructions. Assay reference ranges are outlined in Table 1.

**Table 1:** Clinical, biochemical and densitometric factors between acute (*n* = 11) and chronic (*n* = 9) TSCI cohorts at study baseline

Parameter (mean ± SD, or %)	Interventional acute TSCI cohort (*n* = 11)	Non-interventional chronic TSCI cohort (*n* = 9)	*P*-value
Age at enrolment (years)	39.8 ± 15.8	36.7 ± 16.2	0.666
Sex (% male)	91%	67%	0.285
Ethnicity (% Caucasian)	82%	89%	0.660
Weight (kg)	78.2 ± 14.9	77.5 ± 13.4	0.910
BMI (kg/m^2^)	24.0 ± 4.7	24.7 ± 5.5	0.774
Alcohol (% current)	91%	89%	1.000
Smoking (% current)	9%	11%	0.544
Motor-complete lesion (% AIS grade A or B)	55%	78%	0.279
Level of injury (cervical)	73%	44%	0.199
Level of injury (thoracic)	27%	56%	0.199
Time between SCI and baseline assessment (days)	63 ± 15 (35–83)	1007 ± 549 (370–1733)	< 0.001
Corrected calcium (mmol/L)	2.47 ± 0.19	2.38 ± 0.08	0.173
Phosphate (mmol/L)	1.51 ± 0.16	1.19 ± 0.12 (*n* = 8)	< 0.001
Magnesium (mmol/L)	0.82 ± 0.11	0.82 ± 0.04	0.964
Creatinine (umol/L)	60.8 ± 6.6	65.9 ± 20.2	0.488
25-hydroxyvitamin D_3_ (nmol/L)	78.5 ± 24.8	79.6 ± 31.3	0.937
PTH (pmol/L)	3.4 ± 1.2 (*n* = 10)	4.9 ± 2.8	0.149
ALP (U/L)	101.4 ± 35.5	79.4 ± 19.2	0.114
TSH (mIU/L)	1.5 ± 1.1	1.1 ± 0.5	0.363
Testosterone (nmol/L)	13.5 ± 5.6 (*n* = 10)	19.7 ± 4.6 (*n* = 6)	0.017
LH (IU/L)	3.8 ± 1.5	3.7 ± 1.1	0.851
CTx (ng/L)	1533 ± 645	627 ± 319	< 0.001
P1NP (ug/L)	164 ± 75	75 ± 34	0.002
Sclerostin (ng/L)	279 ± 75	156 ± 69	0.001
Myostatin (ng/L)	1944 ± 1053	1612 ± 690	0.408
Lumbar spine BMD (g/cm^2^)	1.17 ± 0.15 (*n* = 9)	1.05 ± 0.16 (*n* = 8)	0.123
Lumbar spine T-score (SD)	0.7 ± 1.4 (*n* = 9)	− 0.4 ± 1.4 (*n* = 8)	0.132
Lumbar spine Z-score (SD)	1.0 ± 1.4 (*n* = 9)	− 0.1 ± 1.7 (*n* = 8)	0.172
Left femoral neck BMD (g/cm^2^)	0.93 ± 0.19	0.70 ± 0.15	0.006
Left femoral neck T-score (SD)	− 0.1 ± 1.3	− 1.7 ± 1.2	0.013
Left femoral neck Z-score (SD)	0.5 ± 1.2	− 1.1 ± 1.3	0.022
Left total hip BMD (g/cm^2^)	1.03 ± 0.18	0.77 ± 0.16	0.003
Left total hip T-score (SD)	0.1 ± 1.1	− 1.6 ± 1.3	0.007
Left total hip Z-score (SD)	0.4 ± 1.1	− 1.2 ± 1.3	0.018
Left distal femoral epiphysis BMD (g/cm^2^)	1.10 ± 0.28 (*n* = 9)	0.73 ± 0.19 (*n* = 7)	< 0.001
Left distal femoral metaphysis BMD (g/cm^2^)	0.99 ± 0.27 (*n* = 9)	0.76 ± 0.23 (*n* = 7)	0.030
Left proximal tibial epiphysis BMD (g/cm^2^)	0.98 ± 0.26 (*n* = 9)	0.69 ± 0.15 (*n* = 7)	0.004
Total body fat percentage (%)	30.7 ± 6.5 (*n* = 9)	36.1 ± 8.6	0.153
Skeletal muscle index (kg/m^2^)	9.4 ± 7.6 (*n* = 9)	6.2 ± 1.4	0.229

Normal-range for laboratory parameters are as follows: corrected calcium (2.10–2.60 mmol/L), phosphate (0.75–1.50 mmol/L), magnesium (0.70–1.10 mmol/L), 25OHvitaminD_3_ (50–140 nmol/L), PTH (1.6–7.2 pmol/L), ALP (30–110 U/L), TSH (0.4–4.0 mIU/L), testosterone (8.0–30.0 nmol/L), LH (0.6–12.1 IU/L), CTx (50–800 ng/L), P1NP (15-–90 ug/L), sclerostin (124–853 ng/L), myostatin (394–8,018 ng/L)

*TSCI* traumatic spinal cord injury, *SD* standard deviation, *BMI* body mass index, *AIS* American Spinal Injury Association Impairment Scale, *PTH* parathyroid hormone, *ALP* alkaline phosphatase, *TSH* thyroid stimulating hormone, *LH* luteinising hormone, *CTx* C-terminal telopeptide of type 1 collagen, *P1NP* procollagen type 1 N-propeptide, *BMD* body mass index

### BMD Measurements

Participants also underwent DXA scans (Hologic Inc., Waltham, MA, USA) to assess areal BMD in g/cm^2^ of lumbar spine (at least two interpretable vertebrae without hardware or grossly spuriously elevated BMD), left femoral neck, left total hip/proximal femur, left knee (including proximal tibia and distal femur) and whole-body composition (total body fat (%) and skeletal muscle index (kg/m^2^). BMD *T*-scores (SD) and *Z*-scores (SD) at lumbar spine and proximal femoral sites were recorded compared to a young sex-matched and age-matched Caucasian reference population, respectively. The DXA machine was calibrated daily using a Hologic spine phantom. All scans were performed on the same densitometer and analysed by the same licenced investigator. BMD assessment of lumbar spine, left total hip, femoral neck and whole-body composition analyses were performed according to standard operating procedures.

### Protocol for Knee DXA Measurement

**Fig. 2:**
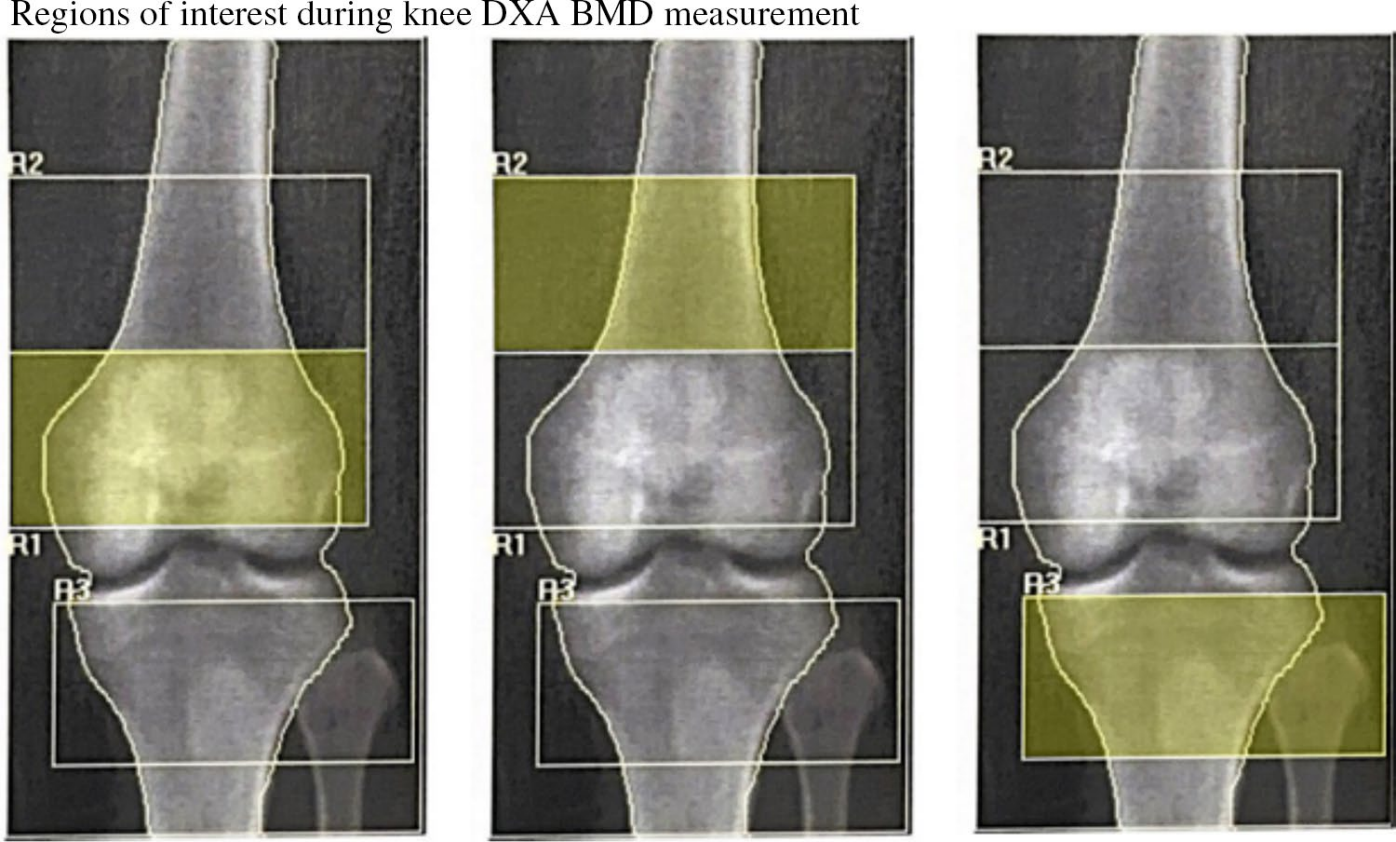
R1 (region of interest 1) = 0–10% distal femur (femoral epiphysis); R2 (region of interest 2) = 10–20% distal femur (femoral metaphysis); R3 (region of interest 3) = 0–10% proximal tibia (tibial epiphysis).

An institutional protocol for performing knee DXA BMD scans was developed and adapted from prior protocols [19, 20]. After discussion with Hologic, the prosthetic hip scan protocol was elected for knee BMD measurement. Whilst supine, participants had their left leg adequately positioned: leg stabilised in full extension, and foot internally rotated, and strapped in a positioner [19]. After performing the initial scan, the participants’ leg was repositioned (without alighting from the bed) and restabilised and scans repeated to obtain two measurements at each scheduled BMD assessment to facilitate precision measurement. Three skeletal regions of interest (ROIs) [20] were analysed, including R1 (0–10% distal femur), R2 (10–20% distal femur), and R3 (0–10% proximal tibia) (Fig. 2). These regions corresponded to the distal femoral epiphysis, distal femoral metaphysis and proximal tibial epiphysis, respectively. Segment lengths were derived from bedside measurement of femur and tibia length. Measures of knee BMD precision error including coefficient of variation (CV, %) and least significant change (LSC) at 95% confidence level (g/cm^2^) were determined using the International Society of Clinical Densitometry calculator, available at: https://iscd.org/learn/resources/calculators/precision-calculator/. CV and LSC at R1, R2 and R3 were 1.8%, 2.7% and 3.1%, and 0.044 g/cm^2^, 0.063 g/cm^2^, and 0.055 g/cm^2^, respectively, indicating a good degree of precision consistent with other published protocols [19, 20].

### Outcomes

Primary outcomes were percentage change in DXA BMD values at lumbar spine, left total hip, left femoral neck, left distal femoral epiphysis, left distal femoral metaphysis and left proximal tibial epiphysis at 12 months within both cohorts. Secondary outcomes included 6-monthly changes in serum bone turnover markers (CTx, P1NP), 12-monthly changes in BMD values, and incidence of fractures, which were recorded at each 6-monthly visit and identified either by an established electronic medical record fracture screening tool, self-reporting or documented fracture-related admission.

### Statistical analyses

Comparison of baseline parameters between groups were analysed using Chi-square test for categorical variables and two-tailed independent samples *t*-test for continuous variables. Within-group longitudinal changes in BMD values and bone turnover marker concentrations were assessed using paired samples *t*-test. Distribution of categorical variables were displayed as frequencies (%) and continuous variables as mean ± standard deviation. Pearson’s correlations were performed to analyse relationships between variables at baseline. A *p*-value of < 0.05 was considered statistically significant. Statistical analyses were conducted using IBM® SPSS®, Version 28.0 (SPSS Inc., Chicago, IL, USA).

## Results

### Baseline Musculoskeletal Parameters in Both Cohorts

Eleven and nine patients, respectively, were recruited in the acute and chronic TSCI cohorts. The total cohort (*n* = 20) was predominantly male (80%) and Caucasian (85%). Majority had motor-complete (65%) and cervical-level injuries (60%). Mean time elapsed between TSCI and baseline assessment was 63 ± 15 days in the acute cohort and 1007 ± 549 days in the chronic cohort (*p* < 0.001). Otherwise there were no significant between-group differences in demographic, patient- or injury-related factors (Table 1). At baseline assessment, acute TSCI patients had higher mean serum phosphate (1.51 ± 0.16 mmol/L vs 1.19 ± 0.12 mmol/L, *p* < 0.001) although corrected calcium, 25OHD_3_, PTH and ALP concentrations did not differ. Mean serum testosterone concentrations were lower in men in the acute cohort (13.5 ± 5.6 nmol/L vs 19.7 ± 4.6 nmol/L, *p* = 0.017) with no difference in LH. Two patients with acute TSCI had baseline testosterone values below the reference range which normalised at 6-months. Mean serum CTx and P1NP were both elevated in acute TSCI patients (1533 ± 645 ng/L vs 627 ± 319 ng/L, *p* < 0.001 and 164 ± 75 ug/L vs 75 ± 34 ug/L, *p* = 0.002, respectively). Baseline serum CTx and P1NP strongly correlated with each other (*r* = 0.746, *p* < 0.001). Mean serum sclerostin concentrations were elevated in acute TSCI (279 ± 75 vs 156 ± 69 ng/L, *p* = 0.001). At study baseline, lumbar spine BMD was preserved in chronic compared with acute TSCI, however, there were lower mean BMD values at left femoral neck (24.7%), total hip (25.2%), distal femoral epiphysis (33.6%), distal femoral metaphysis (23.2%) and proximal tibial epiphysis (29.6%). Three patients were excluded from lumbar spine BMD analyses due to spinal hardware. Three of nine chronic TSCI patients had evidence of osteoporosis at study baseline (*T*-score ≤ -2.5 SD at lumbar spine, total hip and/or femoral neck). Body composition outcomes did not differ between groups.

Shorter time since injury strongly correlated with higher phosphate, CTx, P1NP, sclerostin and lower PTH concentrations, while longer duration of injury correlated with lower left hip and left knee BMD (Supp. Table 1).

### Follow-Up Data in the Acute Interventional TSCI Cohort

Median follow-up for bone turnover marker data were 42 months in the acute TSCI cohort (*n* = 8 had data up to 36 months). Median follow-up duration for BMD data were 48 months (*n* = 7 had data to 48 months and *n* = 2 had no follow-up BMD performed). Two patients were lost to follow-up at 18 months and 24 months, respectively. No fractures occurred in the acute TSCI cohort. Six patients were ambulant (with or without the assistance of mobility aids) by 12 months, of whom the majority had motor-incomplete SCI at baseline (5/6; 83%).

**Fig. 3:**
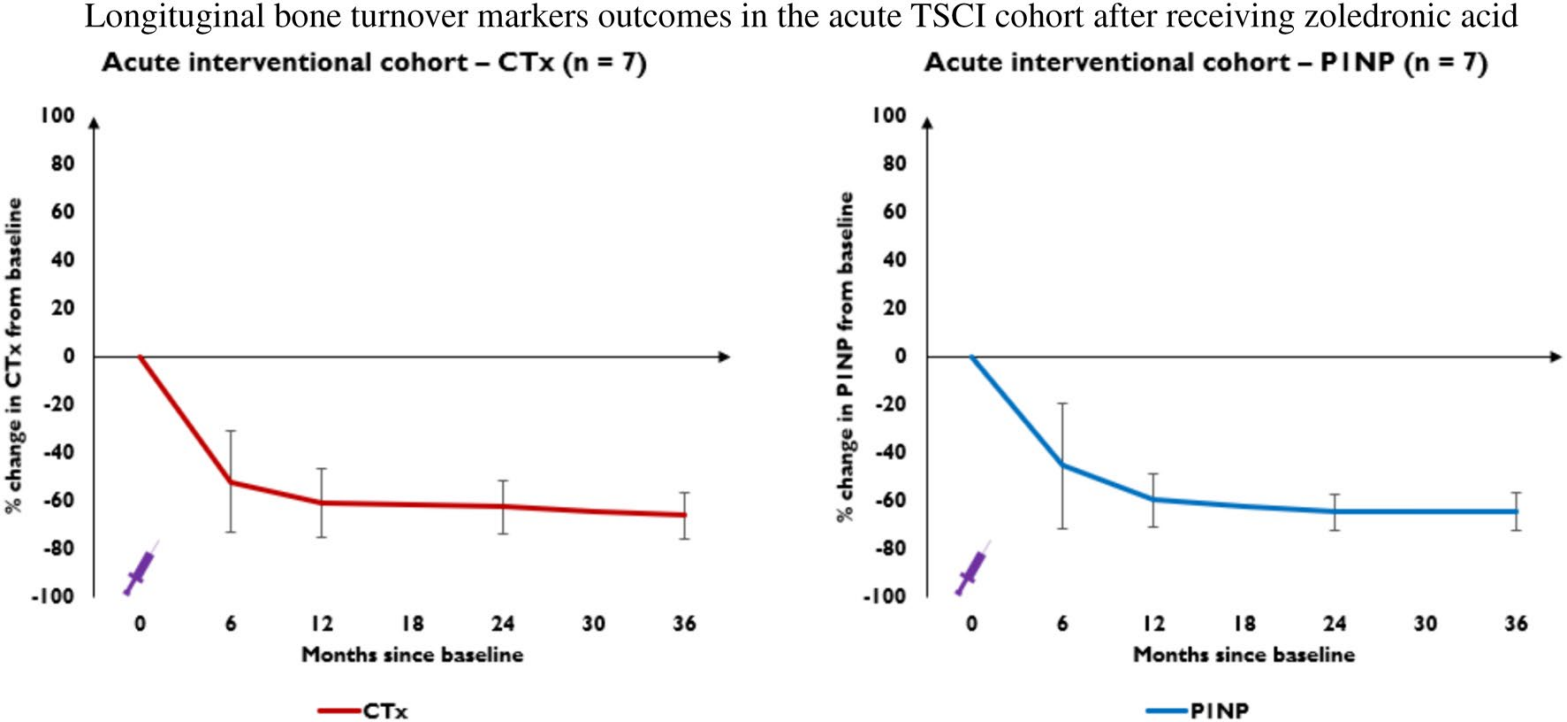
This figure represents percentage change from baseline in serum CTx (red line) and P1NP (blue line) concentrations at 6-, 12-, 24- and 36-months in the acute interventional TSCI cohort. The purple syringe represents the infusion of 4 mg IV zoledronic acid patients received at baseline

Seven patients in the acute TSCI cohort had bone turnover marker data beyond 24 months. Serum CTx declined by mean 52.3% at 6 months (*p* = 0.006) and plateaued at 66.3% by 36 months (*p* = 0.002), while P1NP concentration declined by mean 45.6% at 6 months (*p* = 0.017) and plateaued at 64.8% reduction by 36 months (*p* = 0.001) (Fig. 3). CTx and P1NP concentrations decreased significantly from baseline at each time-point, however, changes between 6-, 12-, 24- and 36 months were not significant (data not shown).

**Fig. 4:**
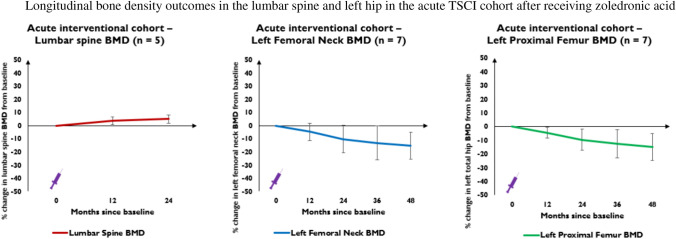
This figure represents percentage change from baseline in lumbar spine BMD (red line), left femoral neck BMD (blue line) and left proximal femur/total hip BMD (green line) at 12-, 24-, 36- and 48-months in the acute interventional TSCI cohort. The purple syringe represents the infusion of 4 mg IV zoledronic acid patients received at baseline

Five patients with acute TSCI had lumbar spine BMD data to 24 months, during which BMD increased non-significantly by mean 5% (*p* = 0.053) (Fig. 4). Seven patients (left hip) and six patients (left knee) had lower limb BMD data beyond 24 months. Despite receiving ZOL at baseline, patients experienced rapid mean BMD decline at 12-, 24-, 36- and 48 months in left femoral neck by 4.7% (*p* = 0.086), 10.2% (*p* = 0.043), 13.1% (*p* = 0.034) and 15.2% (*p* = 0.011) and proximal femur by 4.4% (*p* = 0.038), 9.6% (*p* = 0.027), 12.7% (*p* = 0.025) and 14.9% (*p* = 0.015), respectively (*n* = 7) (Fig. 4). Left hip BMD losses were significant between all 12monthly time-points except 12–24 months and 24–36 months. At conclusion, one patient in the acute cohort developed osteoporotic *T*-scores ≤  − 2.5 SD at the total hip approximately 3-years post-injury.

**Fig. 5:**
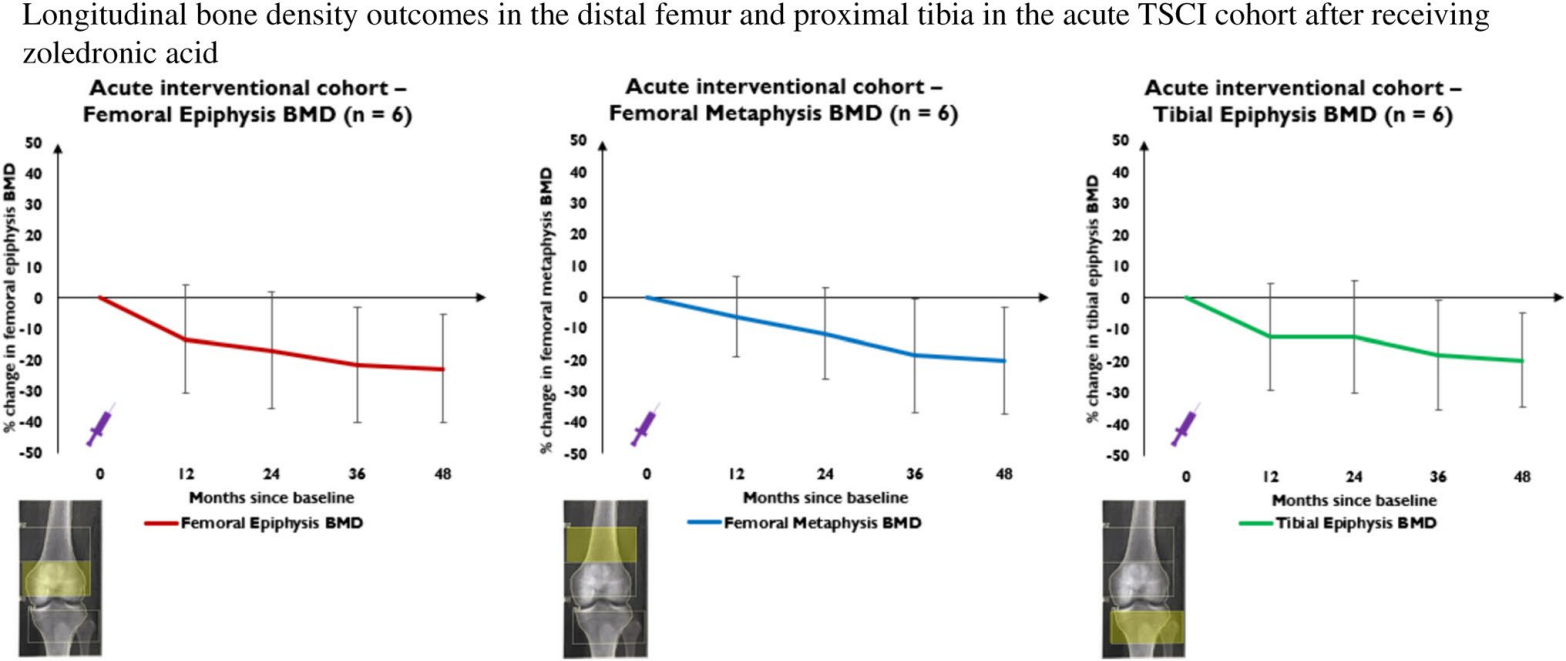
This figure represents percentage change from baseline in left femoral epiphyseal BMD (red line), femoral metaphyseal BMD (blue line) and tibial epiphyseal BMD (green line) at 12-, 24-, 36- and 48-months in the acute interventional TSCI cohort. The purple syringe represents the infusion of 4 mg IV zoledronic acid patients received at baseline

Losses were observed in left knee BMD across all three ROIs at 12-, 24-, 36- and 48 months, in R1 by 13.2% (*p* = 0.134), 17% (*p* = 0.081), 21.6% (*p* = 0.045), 22.8% (*p* = 0.031), R2 by 6.3% (*p* = 0.239), 11.6% (*p* = 0.123), 18.7% (*p* = 0.067) and 20.3% (*p* = 0.051) and R3 by 12.3% (*p* = 0.175), 12.4% (*p* = 0.183), 18.2% (*p* = 0.081), 19.6% (*p* = 0.05), respectively (*n* = 6) (Fig. 5).

**Fig. 6:**
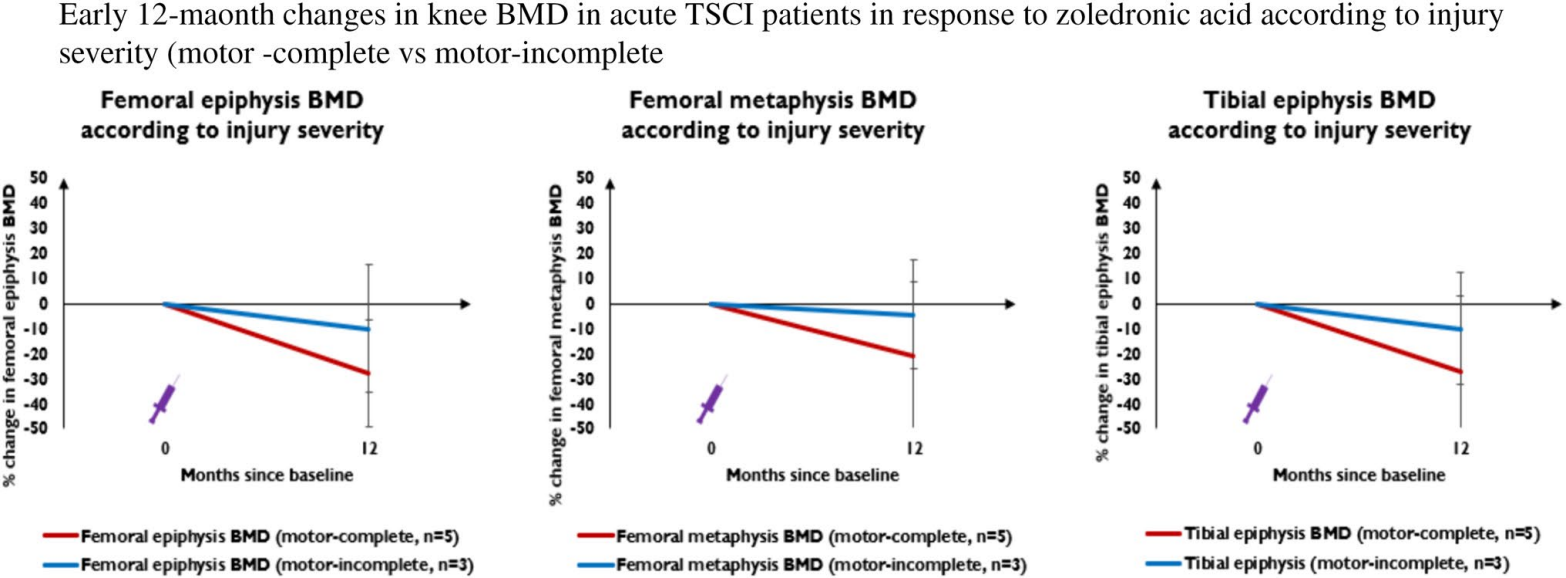
This figure represents percentage change from baseline in left femoral epiphyseal BMD, femoral metaphyseal BMD and tibial epiphyseal BMD at 12-months in acute SCI patients with motor-complete (red lines) and motor-incomplete lesions (blue lines). The purple syringe represents the infusion of 4 mg IV zoledronic acid patients received at baseline

To assess whether extent of impairment influenced early bone turnover marker and BMD responses to ZOL in acute TSCI, participants were grouped into those with motor-complete (*n* = 6) and motor-incomplete lesions (*n* = 5). No between-group difference was observed regarding decline in serum CTx, P1NP concentrations, left femoral neck and total hip BMD in the first 12 months (data not shown). However, there was greater, albeit non-significant, knee BMD loss over 12 months in all three ROIs in patients with motor-complete (*n* = 5) vs motor-incomplete lesions (*n* = 3) (Fig. 6).

APRs occurred in 9/11 (82%) acute TSCI patients receiving ZOL. All APR episodes were of grade II severity and median duration was 2 days (range 1–4 days). The most common symptoms included fever (*n* = 8), rigors (*n* = 7) and generalised musculoskeletal discomfort (*n* = 4). All patients received 25 mg/day oral prednisone for 3-days after symptom onset.

### Follow-Up Data in the Chronic Non-interventional TSCI Cohort

Median follow-up for bone turnover marker data were 36 months in the chronic TSCI cohort (*n* = 6 had data to 36 months). Median follow-up for BMD data were 36 months (*n* = 6 had data to 36 months and *n* = 1 had no follow-up BMD). One patient was lost to follow-up at 12 months. One patient experienced a traumatic right ulnar fracture after a fall during follow-up. Longitudinal changes in bone turnover markers and BMD at 36- and 48 months were not analysed due to insufficient data. From baseline to 24 months, serum CTx declined non-significantly by mean 10.3% (*p* = 0.418) and P1NP by 33.8% (*p* = 0.292) (*n* = 5). Left femoral neck BMD declined by mean 3.4% (*p* = 0.546) with no change in left proximal femur BMD (*n* = 5). There was insufficient data to analyse longitudinal changes over 24-months in lumbar spine, distal femur and proximal tibia BMD values. Longitudinal bone turnover marker and BMD data were not compared between the chronic and acute TSCI cohorts due to inherent differences in injury onset.

## Discussion

In this open-label non-randomised study, early musculoskeletal assessment was performed in patients with acute TSCI within 12 weeks of injury (compared with chronic TSCI patients 1–5 years post-injury). The acute cohort received early ZOL at baseline. Acute and chronic TSCI patients demonstrated different skeletal phenotypes at baseline, including higher markers of bone turnover and sclerostin concentrations in the acute cohort and mean ~ 20–30% lower hip and knee BMD in the chronic cohort. Despite early ZOL, acute TSCI patients experienced rapid mean ~ 15% hip BMD losses and even greater declines of ~ 20% at the knee over 4 years. BMD losses after acute TSCI led to osteoporosis in one patient although no fragility fractures occurred during follow-up. Preliminary data, albeit not significant, suggested more severe impairment (i.e., motor-complete lesion) may predict worse knee BMD response in the first 12 months after ZOL. APRs occurred frequently in acute TSCI patients receiving ZOL. Longitudinal assessment of the ‘natural history’ of osteoporosis in chronic TSCI patients was limited by insufficient data.

Patients with acute TSCI had higher serum phosphate concentrations. This is consistent with prior studies demonstrating higher serum phosphate and calcium and urinary calcium excretion and lower PTH in the first 6–12 months post-TSCI, potentially related to the exaggerated bone resorption [4, 6, 21, 22]. Although serum calcium was similar in our two cohorts, we did not assess ionised calcium or urinary calcium concentration, which may have revealed more subtle changes in calcium homeostasis. Acute TSCI patients in our study had higher serum markers of bone turnover, reflecting an exaggerated state of bone turnover in the acute state. It was previously suggested an uncoupling of bone resorption and formation may occur acutely after TSCI based on observations of markedly elevated bone resorption (e.g., deoxypyridinoline) and normal/mildly elevated bone formation markers (e.g., osteocalcin, total and bone-specific ALP) [4, 6, 22–24]. However, more recently, both CTx and P1NP have been shown to be elevated in acute SCI. A cross-sectional study found acute TSCI patients (*n* = 24) had CTx and P1NP concentrations at least double those in chronic TSCI (*n* = 38) and that higher concentrations correlated with shorter duration since injury [25]. Prospective studies have consistently shown CTx and P1NP are elevated in acute TSCI [26–29] with one study demonstrating a significant decline of P1NP over 24 months post-TSCI whilst b-ALP remained stable [26]. Similarly, in our study, baseline serum CTx and P1NP both correlated with shorter time since injury and strongly correlated with each other and hence may be more sensitive markers of bone turnover in acute TSCI. Small, randomised placebo-controlled studies have demonstrated a greater decline in bone turnover markers at 3–6 months post-injury in ZOL-treated patients, however, no difference at 1 year, suggesting earlier bone turnover marker response may be a useful marker of antiresorptive treatment effect in acute TSCI [26–29]. Although our study lacked a control group, our results are consistent since despite experiencing mean ~ 60% decline in CTx and P1NP within 1 year of receiving ZOL, patients with acute TSCI still lost considerable bone mass. The proportion of exaggerated bone turnover in acute SCI secondary to dysregulated skeletal homeostasis vs fracture healing is unclear. A retrospective study showed higher CTx in acute TSCI patients who sustained fractures during their injury or required skeletal surgery [30].

At study baseline, patients with acute TSCI had higher serum sclerostin concentrations. Studies consistently show sclerostin concentrations are highest in the acute phase and correlate with shorter time since injury during the first 5-years [25, 31]. Sclerostin elevations likely reflect dysregulated osteoblastic signalling pathways after acute skeletal unloading. Sclerostin (encoded by *SOST* gene) is an osteocyte-derived inhibitor of Wnt signalling and hence diminishes bone formation [32]. Osteocytes respond to skeletal loading-induced mechanical strain by downregulating sclerostin expression and triggering increased bone formation (*mechanotransduction*) [32]. The importance of sclerostin to skeletal homeostasis is well-established, and romosozumab (anti-sclerostin monoclonal antibody) has emerged as a potent osteoanabolic agent in osteoporosis treatment [32, 33]. In chronic TSCI, sclerostin concentrations correlate with knee and hip BMD rather than time since injury and hence lower sclerostin levels may reflect increasing osteoporosis severity [31, 34, 35]. No studies have explored longitudinal changes in sclerostin concentrations post-TSCI, and hence the timing of sclerostin peak is unclear. Data are also lacking for whether circulating sclerostin concentrations reflect osseous sclerostin expression. De Mare et al. showed serum sclerostin moderately correlated with osseous sclerostin expression in patients with end-stage renal failure (*n* = 68) [36]. However, sclerostin concentrations (including with the DiaSorin assay) are elevated in renal impairment (unpublished data) which may have impacted these results. There is emerging interest in the use of romosozumab for post-TSCI bone loss, with studies in acute (NCT04597931) and chronic SCI (NCT05101018, NCT04232657) recruiting.

At baseline, chronic TSCI patients had lower BMD in femoral neck and total hip with between-group differences more pronounced for distal femur and proximal tibia BMD, whilst lumbar spine BMD was relatively spared. Lower BMD values in the proximal femur, distal femur and proximal tibia all correlated with longer time since injury. These results are consistent with other studies comparing BMD between acute and chronic SCI cohorts. Duration since injury is an established risk factor for BMD loss after SCI, as patients have more prolonged exposure to sub-lesional bone loss [4, 6, 23, 25].

Acute phase reactions (APRs) occurred in all but two patients in our cohort of acute TSCI patients receiving ZOL (9/11; 82%) despite prophylactic paracetamol [38] although no cases were severe or prolonged. APRs occur commonly in ~ 40% of postmenopausal women with osteoporosis receiving their first dose of ZOL [17]. The high rate of APRs in our acute TSCI cohort are consistent with other such studies (pooled incidence 69% (55/80)), albeit APRs being heterogeneously defined [24, 26, 28, 29, 37] (Supp. Table 2). The underlying mechanism for seemingly high rates of APR after acute TSCI has not been elucidated. In our cohort, given almost all patients had an APR (all grade II), we were unable to examine risk factors for presence and severity of APR. Exaggerated bone turnover in acute TSCI may play a role. Higher concentrations of bone turnover markers (P1NP, CTx), although only modestly, have been associated with increased APR risk in cohorts of predominantly postmenopausal women [39, 40]. The acute inflammatory state of TSCI may also contribute, given the immune mechanism for APR and association with higher inflammatory markers [41, 42]. However, inflammatory markers have not routinely been assessed in acute TSCI patients receiving ZOL. Acute TSCI cohorts are typically young and although younger age was shown to be a risk factor in postmenopausal osteoporosis, this was in the range of early-60's to late-70's [17, 39]. Patients with acute TSCI typically are male, with higher BMD at time of injury and no prior oral bisphosphonate exposure, and it is unclear whether these may further contribute to increased APR risk [17, 39–41, 43].

Despite early ZOL in our acute TSCI cohort, rapid BMD loss still occurred at the hip and particularly at the knee. Conclusions regarding efficacy of ZOL in preventing BMD loss are however limited in the absence of controls with acute TSCI not receiving ZOL. However, given patients still lost a mean 15% BMD in the hip and 20% in the knee over 4-years, it is unlikely any benefit of ZOL was clinically meaningful in preventing BMD loss. Our results are consistent with prior small prospective controlled trials which utilised a single infusion of ZOL in the first 3–4-months post-TSCI with majority of studies limited to 1-year follow-up [24, 26–29, 37, 44] (Supp. Table 2). Participants experienced some attenuation of hip BMD (net difference of ~ 10–15% vs controls) one year after ZOL, however, still lost ~ 5–10% of BMD, suggesting more effective strategies are needed to completely attenuate bone loss. A second annual infusion of ZOL was utilised in an exploratory extension of one study which completely attenuated BMD loss at the total hip and femoral neck, and thus a strategy of two annual infusions of ZOL early in acute TSCI warrants further investigation.

At time of study conceptualisation, only two studies had reported knee BMD outcomes after ZOL in acute TSCI with disappointing results (Supp. Table 2). Bauman et al. assessed thirteen patients with acute TSCI managed with (*n* = 6) and without (*n* = 7) early ZOL [37]. The treatment group experienced significantly greater BMD losses compared to controls at the distal femur and proximal tibia after 12 months, unexplained by low precision. However, the controls in this study, despite all having motor-complete TSCI, had far less pronounced knee BMD losses than expected in the first year post-TSCI. Schnitzer et al. conducted a randomised placebo-controlled trial in seventeen patients with acute TSCI [28]. A single infusion of ZOL led to 5–10% better knee BMD responses at 6 months, however patients still experienced mean total BMD loss > 20% at the knee after 2-years. A recent study supported the early but short-lasting effect of ZOL on preventing knee BMD loss, with a difference of 6–8% at 4 months favouring ZOL but minimal-to-no difference by 12 months [27]. A larger placebo-controlled study (*n* = 60) utilised CT-derived BMD assessment of the distal femur and proximal tibia and similarly showed partial attenuation of cortical and trabecular BMD losses at 12 months but dramatic BMD losses in both groups at 24 months [26]. Collectively, these studies suggest a single infusion of ZOL may help prevent some bone loss at the knee in the first 6 months post-TSCI, but that the benefit is almost all lost as early as 2-years. Unlike with hip BMD, two annual ZOL infusions were unable to prevent dramatic declines in CT-derived compartmental knee BMD [25]. Given the importance of knee BMD assessment in TSCI, we developed an institutional protocol for this measurement which demonstrated a good degree of precision. Our study is the first to report BMD outcomes beyond 2 years in patients receiving ZOL in acute TSCI and demonstrated ongoing rapid bone loss around the knee in the 3rd and 4th years post-injury at a similar rate to that seen in the first 2 years. Our results, despite absence of a control group, are consistent with previous studies demonstrating lack of meaningful knee BMD response to a single ZOL infusion and indicate other treatment approaches need to be investigated. The use of short-term 6-monthly denosumab has been shown to prevent early hip and knee BMD loss in acute TSCI. However ~ 10–15% BMD losses occurred within 12 months of subsequent denosumab cessation, and hence this may not be a suitable strategy particularly given the younger age of acute TSCI cohorts [45].

In prior studies evaluating ZOL for preventing acute bone loss after TSCI, there was no delineation regarding clinical or pathological factors which may predict a better (or worse) BMD response. Complete motor impairment below the lesion is a marker of lesional severity and is a consistent risk factor for greater bone loss and fracture risk post-TSCI [3, 9, 10]. Despite this, our study is the first to perform an analysis comparing BMD responses to ZOL according to lesional severity. Our data suggests patients with motor-complete lesions tend to have greater BMD losses in the proximal tibia and distal femur after early ZOL. Patients with motor-complete lesions may represent a ‘high-risk’ cohort who require more intensive early pharmacotherapy to prevent acute bone loss. Despite a seemingly large numerical difference, this result is hypothesis-generating only and warrants further exploration in larger longitudinal studies.

Our study possesses various limitations. There was no direct control group for the acute interventional TSCI cohort as despite the non-interventional cohort not receiving ZOL, these patients were recruited much later after their injury. Given there had been some placebo-controlled evidence published prior to study conceptualisation that a single infusion of ZOL can attenuate some BMD loss at the hip in acute TSCI, we deemed it unethical to randomise our cohort to receive placebo, particularly given this study aimed to assess outcomes over a longer four year period. In our study, a cohort of patients with chronic TSCI were included to facilitate baseline comparisons in musculoskeletal parameters and also to assess the natural history of bone turnover and bone loss in this cohort. Adjunctive rehabilitation procedures were not recorded and ambulatory status of patients was not formally assessed, which would have allowed more detailed characterisation of our cohort. Participant recruitment was limited by constraints of the COVID-19 pandemic and geographical barriers as Royal North Shore Hospital is a tertiary referral centre for patients with TSCI including from regional and rural New South Wales. There were instances of missed BMD assessments (e.g., due to loss to follow-up, geographic limitations, and musculoskeletal discomfort) which further limited power in longitudinal and subgroup analyses (particularly in the chronic TSCI cohort). Several patients had exclusion of lumbar spine BMD for analysis due to spinal surgery post-TSCI, although this is a less relevant site in TSCI-related osteoporosis. The study is strengthened by protocolised and precise assessment of BMD at the knee which is lacking in several associated studies despite its demonstrated clinical relevance in TSCI-related osteoporosis. Comprehensive baseline clinical, biochemical and densitometric data allowed exploration of key musculoskeletal differences between acute and chronic TSCI patients. We were able to assess durability of response to a single infusion of ZOL in acute TSCI up to 4 years, with such an extended follow-up not previously reported.

## Conclusion

An effective method to prevent early profound bone loss in the hip and particularly the knee after acute TSCI remains elusive. This prospective study further characterises the unique musculoskeletal characteristics of patients early after sustaining TSCI. Although our study is limited by lack of a control group, our results support previous findings of early dramatic BMD losses in the hip and knee in the first 1–2-years post-TSCI despite a single infusion of ZOL. Our findings are novel as we demonstrate ongoing BMD losses over a longer follow-up period up to 4 years after ZOL in acute TSCI. Our exploratory analyses generate the novel hypothesis that motor-complete TSCI may predict a worse knee BMD response to ZOL. Whether such patients represent a ‘high-risk’ cohort requiring more intensive early pharmacotherapy to curb acute bone loss warrants further investigation. All future studies assessing skeletal responses to osteoporosis treatment in acute TSCI must incorporate precise longitudinal assessment of knee BMD; the most clinically relevant site for bone loss and fracture risk. Follow-up should extend beyond 1–2 years to help determine whether other strategies beyond a single early infusion of ZOL (e.g., repeated infusions or osteoanabolic pharmacotherapy) may prevent bone loss in the first few years post-TSCI. Establishing an effective strategy for preventing profound early bone losses post-TSCI would dramatically reduce lifetime risk of osteoporosis and fragility fractures in this priority population.

## Supplementary Information

Below is the link to the electronic supplementary material.

Supplementary file1 (DOCX 17 KB)

Supplementary file2 (DOCX 19 KB)

## Declarations

### Research Involving Human and Animal Participants

All procedures performed in this study involving human participants were in accordance with the ethical standards of the 1964 Helsinki declaration and its later amendments or comparable ethical standards. Ethics approval was obtained from the Northern Sydney Local Health District (NSLHD) Human Research Ethics Committee (2019/ETH09718).
