# Supplementary material for: Preventing OsteoPorosis in Spinal Cord Injury (POPSCI) Study—Early Zoledronic Acid Infusion in Patients with Acute Spinal Cord Injury
Source: Calcif Tissue Int. 2024 Sep 25;115(5):611–23. doi: 10.1007/s00223-024-01292-3 (PMC11531416; doi:10.1007/s00223-024-01292-3)
Supplement: Supplementary file 1 — Supplementary file1 (DOCX 17 KB) [file 223_2024_1292_MOESM1_ESM.docx]

**Supplemental Table 1 – Pearson’s correlations between time since injury and baseline musculoskeletal parameters**

| **Parameter** | **Correlation coefficient (r)** | **P-value** |
| --- | --- | --- |
| Serum corrected calcium | -0.193 | 0.414 |
| Serum phosphate | -0.685 | 0.001 |
| Serum ALP | -0.332 | 0.153 |
| Serum PTH | 0.486 | 0.035 |
| Serum testosterone | 0.440 | 0.088 |
| Serum CTx | -0.578 | 0.008 |
| Serum P1NP | -0.480 | 0.032 |
| Serum sclerostin | -0.476 | 0.034 |
| Serum myostatin | -0.016 | 0.946 |
| Lumbar spine BMD | -0.018 | 0.944 |
| Left femoral neck BMD | -0.476 | 0.034 |
| Left total hip BMD | -0.530 | 0.016 |
| Left distal femoral epiphysis BMD | -0.730 | 0.001 |
| Left distal femoral metaphysis BMD | -0.572 | 0.021 |
| Left proximal tibial epiphysis BMD | -0.633 | 0.008 |

ALP = alkaline phosphatase; PTH = parathyroid hormone; CTx = C-terminal telopeptide of type 1 collagen; P1NP = procollagen type 1 N-propeptide; BMD = bone mineral density.
