# Supplementary material for: Preventing OsteoPorosis in Spinal Cord Injury (POPSCI) Study—Early Zoledronic Acid Infusion in Patients with Acute Spinal Cord Injury
Source: Calcif Tissue Int. 2024 Sep 25;115(5):611–23. doi: 10.1007/s00223-024-01292-3 (PMC11531416; doi:10.1007/s00223-024-01292-3)
Supplement: Supplementary file 2 — Supplementary file2 (DOCX 19 KB) [file 223_2024_1292_MOESM2_ESM.docx]

**Supplemental Table 2 – Summary of randomised controlled studies utilising a single infusion of zoledronic acid to prevent bone loss in acute SCI**

| **First author, Year** | **Sample size** | **Time from injury** | **Intervention (n) Control (n)** | **Study endpoints** | **Follow up duration** | **APR rate** | **APR definition** | **Knee BMD (vs controls)** | **Hip BMD (vs controls)** |
| --- | --- | --- | --- | --- | --- | --- | --- | --- | --- |
| Edwards, 2021 | N = 60 | Mean 68 days <4 months | ZOL (n = 30)  Placebo  (n = 30) | DXA BMD  - spine  - hip  CT BMD  - knee | 12 months | 22/30 | Fever (>101^0^F) or  ≥2 symptoms (chills, MSK discomfort, nausea, vomiting, poor appetite, headache) | Attenuated losses in trabecular and cortical BMC and cortical bone volume | +10.6% total hip BMD  +9.6% femoral neck BMD |
| Oleson, 2020 | N = 15 | <3 weeks | ZOL (n = 10)  Placebo  (n = 5) | DXA BMD  - hip - knee  CTx, P1NP | 12 months | 7/10 | Fever (>100.5^0^F) | +1.9% distal femur BMD  +5.5% proximal tibia BMD | +13.1% total hip BMD  +13.1% femoral neck BMD |
| Goenka, 2018 | N = 60 | Mean 27.5 days | ZOL (n = 30)  Standard care  (n = 30) | DXA BMD - hip | 12 months | NR | N/A | N/A | +0.16 g/cm^2^ total hip BMD +0.13 g/cm^2^ femoral neck BMD |
| Schnitzer, 2016 | N = 16 | Mean 69 days | ZOL (n = 7)  Placebo  (n = 9) | DXA BMD - spine - hip - knee  CTx, P1NP | 24 months (intervention)  6 months (control) | 3/7 | Febrile myalgic reaction | +5.4% distal femur BMD vs controls at 6-months  +10.5% proximal tibia BMD vs controls at 6-months  -26.2% distal femur BMD in ZOL group at 24-months -21.9% proximal tibia BMD in ZOL group at 24-months | +14.9% femoral neck BMD  vs controls at 6-months    -4.1% femoral neck BMD in  ZOL group at 24-months |
| Bauman, 2015 | N = 13 | <4 months | ZOL (n = 6)  Standard care  (n = 7) | DXA BMD  - hip  - knee | 12 months | 6/6 | Febrile myalgic reaction | -10.1% distal femur BMD  -12.5% proximal knee BMD | +12.6% total hip BMD  +15.0% femoral neck BMD |
| Bubbear, 2011 | N = 14 | Mean 57 days | ZOL (n = 7)  Standard care  (n = 7) | DXA BMD  - spine - hip - hip geometry  CTx, P1NP | 12 months | 5/7 | Flu-like reaction | N/A | +12.4% total hip BMD  No difference at femoral neck |
| Shapiro, 2007 | N = 17 | Not defined | ZOL (n = 9)  Placebo (n = 8) | DXA BMD  - spine  - hip  b-ALP, urine NTx | 12 months | 3/9 | Febrile myalgic reaction | N/A | No difference at femoral neck |

SCI = spinal cord injury; ZOL = zoledronic acid; DXA = dual-energy X-ray absorptiometry; BMD = bone mineral density; CT = computed tomography; CTx = C-terminal telopeptide of type 1 collagen; P1NP = procollagen type 1 N-propeptide; b-ALP = bone-specific alkaline phosphatase; NTx = N-terminal telopeptide of type 1 collagen; APR = acute phase reaction; NR = not reported; BMC = bone mineral content.
